# Analysis of Phase-Shift Pulse Brillouin Optical Time-Domain Reflectometry

**DOI:** 10.3390/s19071497

**Published:** 2019-03-27

**Authors:** Tsuneo Horiguchi, Yuki Masui, Mohd Saiful Dzulkefly Zan

**Affiliations:** 1 Graduate School of Engineering and Science, Shibaura Institute of Technology, 3-7-5 Toyosu, Koto-ku, Tokyo 135-8548, Japan; ma17105@shibaura-it.ac.jp; 2 Centre of Advanced Electronic and Communication Engineering (PAKET), Faculty of Engineering and Built Environment, Universiti Kebangsaan Malaysia (UKM), Bangi, Selangor 43600, Malaysia; saifuldzul@ukm.edu.my

**Keywords:** Distributed fiber sensor, strain and temperature sensor, Brillouin scattering, Brillouin Optical Time Domain Reflectometry, signal processing, spectral analysis, fast Fourier transform

## Abstract

Distributed strain and temperature can be measured by using local Brillouin backscatter in optical fibers based on the strain and temperature dependence of the Brillouin frequency shift. The technique of analyzing the local Brillion backscatter in the time domain is called Brillouin optical time domain reflectometry (BOTDR). Although the best spatial resolution of classic BOTDR remains at around 1 m, some recent BOTDR techniques have attained as high as cm-scale spatial resolution. Our laboratory has proposed and demonstrated a high-spatial-resolution BOTDR called phase-shift pulse BOTDR (PSP-BOTDR), using a pair of probe pulses modulated with binary phase-shift keying. PSP-BOTDR is based on the cross-correlation of Brillouin backscatter and on the subtraction of cross-correlations obtained from the Brillouin scatterings evoked by each phase-modulated probe pulse. Although PSP-BOTDR has attained 20-cm spatial resolution, the spectral analysis method of PSP-BOTDR has not been discussed in detail. This article gives in-depth analysis of the Brillouin backscatter and the correlations of the backscatters of the PSP-BOTDR. Based on the analysis, we propose new spectral analysis methods for PSP-BOTDR. The analysis and experiments show that the proposed methods give better frequency resolution than before.

## 1. Introduction

Brillouin scattering occurs via the interaction of light with acoustic waves in a medium; this interaction causes a frequency shift, called a Brillouin frequency shift (BFS), due to the Doppler effect. It has been found that the BFS of silica optical fibers increases with longitudinal strain and temperature at a rate of about 0.5 MHz/10 µ-strain and 1 MHz/K, respectively [1]. Additionally, a recent article reported the dependency of the BFS for guided acoustic-wave Brillouin scattering (GAWBS) on the mechanical impedance of substances outside the cladding of optical fibers [2]. Based on the BFS characteristics and spatially-resolved BFS measurement techniques, various types of distributed fiber-optic strain and temperature sensors based on Brillouin scatterings have been reported. They include fiber sensors using backward stimulated Brillouin scattering (SBS) and spontaneous Brillouin scattering (SpBS) [1,3,4,5,6]. SBS-based sensors utilize signals amplified via SBS, and have thus more easily achieved higher resolutions in space and frequency, as well as faster measurements, than SpBS-based sensors. However, SBS-based sensors need to access both ends of the fiber to transmit counter propagating pump and probe lights through the fiber, which makes it difficult to apply SBS-based sensors to cases where light can be launched through only one end of the fiber cable and the fiber cable cannot be folded back at the other end. In contrast to SBS-based sensors, SpBS-based sensors need only one fiber-end access, since the probe pulse and the backscatter can be launched and extracted through the same fiber end. However, spontaneous Brillouin scattering is significantly weaker than the SBS-based signal, and it becomes weaker still if the probe pulse becomes shorter in its duration for realizing higher spatial resolution. Additionally, the Brillouin scattering spectrum (BSS)—measured using a narrower pulse—becomes broader in width, which deteriorates frequency resolution. Therefore, for the classic SpBS-based sensor that uses a short pulse as a probe, called Brillouin optical time domain reflectometry (BOTDR), the best spatial resolution remained around 1 m. However, Brillouin optical correlation domain reflectometry (BOCDR) has broken through this barrier, achieving 40 cm resolution [7] and more recently 6 mm resolution [8]. BOCDR adopts a frequency modulation scheme and transmits the continuous frequency-modulated lightwave through the fiber; the Brillouin backscatter is correlated with the delayed frequency-modulated lightwave via an optical heterodyne detection. Contrary to classic BOTDR, BOCDR can measure the local Brillouin backscatter continuously, and can thus achieve higher signal-to-noise ratio and better spatial resolution than classic BOTDR. However, BOCDR requires that the delay fiber be at least twice as long as the sensing fiber, while the length of the delay fiber should be as short as possible to make fast measurements. This requirement may cause an impediment to the easy and quick operation of BOCDR in practical fields. In contrast, BOTDR needs no delay fiber.

Though not as high in resolution as the BOCDR, recent BOTDRs are gradually making some progress to attain cm-scale resolution by using constructed pulses instead of a common single pulse, and by using special signal processing techniques. These include double-pulse BOTDR [9], differential-technique BOTDR [10], synthetic BOTDR [11] and phase-shift pulse BOTDR (PSP-BOTDR) [12,13]. Among them, double-pulse BOTDR was the first that to attain a cm-scale spatial resolution. Double-pulse BOTDR uses a matched filter to enhance the local Brillouin backscatters due to the double pulse via interference effects. However, unwanted signals from outside the local region are superimposed, although they are small. To remove the unwanted signal, subtraction and cancellation methods have been reported [10,11,12,13]. The differential-technique BOTDR uses a pair of long pulses with a slight difference in width in a way analogous to the differential pulse-width pair Brillouin optical time domain analysis (DPP-BOTDA) [14] based on SBS. Synthetic BOTDR and PSP-BOTDR also employ a set of pulsed probes and employ subtraction techniques to remove unwanted signals. Both BOTDRs apply phase-shift keying modulation to the set of probe pulses; thus, compared to the differential-technique BOTDR, synthetic BOTDR and PSP-BOTDR have the potential to attain double the measurement speed. Synthetic BOTDR employs quadrature phase-shift keying to produce a set of four kinds of probe pulses, while PSP-BOTDR uses binary phase-shift keying, simplifying the modulation and processing. PSP-BOTDR has already been validated in proof of concept experiments, achieving a 20-cm resolution. However, the spectral analysis method used in PSP-BOTDR has not been discussed in detail. This article gives an in-depth analysis of Brillouin backscatter and the correlations of PSP-BOTDR backscatters. Based on the analysis, we propose improved spectral analysis methods for PSP-BOTDR. The analysis and experiments show that the proposed methods give better frequency resolution than the previous one.

## 2. Materials and Methods

### 2.1. Principle of Phase-Shift Pulse Brillouin Optical Time-Domain Reflectometry (PSP-BOTDR)

**Figure 1:**
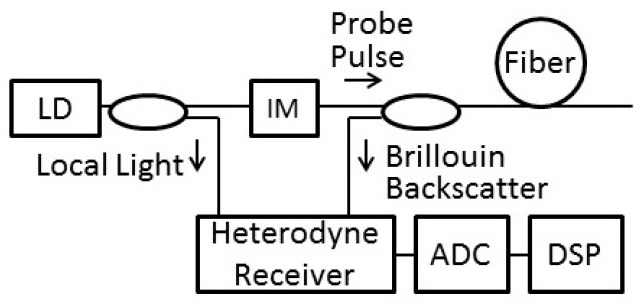
Basic configuration of Brillouin optical time-domain reflectometry (BOTDR). LD: laser diode; IM: intensity modulator; ADC: analog-to-digital convertor; DSP: digital signal processor.

Figure 1 shows a common configuration of BOTDR, where a probe pulse is launched into a sensing fiber; Brillouin backscatter is detected in the time domain by a heterodyne detection scheme with a high sensitivity. If we analyze its spectrum to obtain the BFS, we can map the strain and temperature distribution along the length of the fiber.

We commonly use a single pulse as a probe of BOTDR. Then, we can obtain, for example, a 1-m spatial resolution for a 10-ns pulse as in Rayleigh backscatter-based OTDR. However, if we narrow the pulse width to less than 10 ns to obtain cm-scale spatial resolution, the measured BSS width begins to increase. This is due to the fact that the spectrum width of such a narrow pulse exceeds the intrinsic BSS width, which is determined by the phonon life time for the fiber. This broadening in the measured BSS width deteriorates the frequency resolution of the BOTDR, which is one of the main reasons why it is difficult to achieve cm-scale resolution using classic BOTDR.

**Figure 2:**
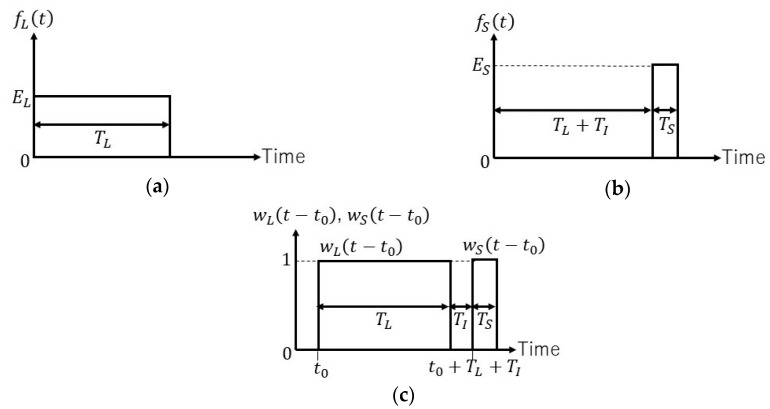
Electric field envelopes and window functions: (**a**) electric field envelope, $f_{L}\left(t\right)$, of a long pulse; (**b**) electric field envelope, $f_{S}\left(t\right)$, of a short pulse; (**c**) a pair of window functions related to long probe pulse and short probe pulse.

To overcome this difficulty, PSP-BOTDR uses a pair of probes. Each probe consists of long and short pulses which are concatenated with and without a short interval [12,13]. The envelopes of the light fields of the long and short pulses, $f_{L}\left(t\right)$ and $f_{S}\left(t\right)$, are shown in Figure 2a,b, respectively, and expressed by

(1) $$\begin{array}{l} f_{L}\left(t\right)=\{\begin{array}{cc} E_{L} & 0<t<T_{L}\\ 0 & t\leq 0,t\geq T_{L} \end{array},\\ f_{S}\left(t\right)=\{\begin{array}{cc} E_{S} & T_{L}+T_{I}<t<T_{L}+T_{I}+T_{S}\\ 0 & t\leq T_{L}+T_{I},t\geq T_{L}+T_{I}+T_{S} \end{array}, \end{array}$$

where $E_{L}$ and $E_{S}$ denote the amplitudes of $f_{L}\left(t\right)$ and $f_{S}\left(t\right)$, and $T_{L}$, $T_{S}$ and $T_{I}$ denote the durations of the long pulse, short pulse and separation between the pulses, respectively.

The short pulse of one probe of the pair is not phase modulated, while the short pulse of the other probe is modulated with π-phase shift. Thus, the light fields ${\tilde{E}}_{in,0}\left(t\right)$ and ${\tilde{E}}_{in,\pi }\left(t\right)$ of the non-phase-shift and π-phase-shift probes can be expressed respectively by

(2) $${\tilde{E}}_{in,0}\left(t\right)=E_{in,0}\left(t\right)e^{j\left(\omega t+\theta_{0}\right)}=\left[f_{L}\left(t\right)+f_{S}\left(t\right)\right]e^{j\left(\omega t+\theta_{0}\right)},$$

(3) $${\tilde{E}}_{in,\pi }\left(t\right)=E_{in,\pi }\left(t\right)e^{j\left(\omega t+\theta_{\pi }\right)}=\left[f_{L}\left(t\right)-f_{S}\left(t\right)\right]e^{j\left(\omega t+\theta_{\pi }\right)},$$

where ω is the optical frequency, and $\theta_{0}$ and $\theta_{\pi }$ are the initial phases.

When using the fast Fourier transform (FFT) to obtain BSS for classic BOTDR with a broadband detector, it is common to directly apply FFT to the backscatter signal sampled with a short window function [15,16]; the absolute square of the FFT result yields the power spectrum of the local Brillouin backscatter. Based on the Wiener–Khinchin theorem, we can also obtain the power spectrum by applying the FFT to the auto-correlation function of the sampled backscatters. So, our first idea to solve the spectrum-broadening problem above is to apply the FFT to the cross-correlation function of the backscatters due to the long and short pulses of the probe [13]. Window functions to sample the backscatters are shown in Figure 2c, having the same durations with the long and short probe pulse, *T_L_* and *T_S_*, and the same interval *T_I_*. Since the absolute value of the FFT of the cross-correlation function gives the product of the absolute values of the FFTs of each windowed signal, the spectrum obtained by this proposed method may be much narrower in width than the BSS obtained with a single short pulse. However, the cross-correlation is not localized. That means that the cross-correlation obtained in the above way is the sum of the cross-correlations of the signals backscattered at different segments in the fiber. So, our next idea is to use a differential technique in conjunction with the cross-correlation. This idea comes from the fact that some SBS-based sensors use a subtraction method to attain cm-scale spatial resolution [17,18]. The subtraction of the cross-correlation obtained using the π-shift probe pulse (Equation (3)) from that of the 0-shift probe pulse (Equation (2)) removes unwanted cross-correlations and only gives the desired cross-correlation between the localized backscatter. Details will be given in the following section.

### 2.2. Differential Cross-Correlation

Consider the spontaneous Brillouin backscatter signals when we launch the probe pulses whose optical fields are expressed by Equations (2) and (3) into a single-mode fiber. For simplicity, we assume that the fiber loss can be ignored. Then, by integrating the light field backscattered at a distance z, we can obtain the backscatter signals detected with a heterodyne receiver at the input end:

(4) $$b_{m,0}\left(t\right)=\int_{0}^{L_{F}}R_{m}\left(t,z\right)E_{in,0}\left(t-\frac{2z}{v}\right)e^{j\left[\omega_{B}\left(z\right)t-\theta_{m}\left(z\right)\right]}dz,$$

(5) $$b_{n,\pi }\left(t\right)=\int_{0}^{L_{F}}R_{n}\left(t,z\right)E_{in,\pi }\left(t-\frac{2z}{v}\right)e^{j\left[\omega_{B}\left(z\right)t-\theta_{n}\left(z\right)\right]}dz,$$

where $b_{m,0}\left(t\right)$ and $b_{n,\pi }\left(t\right)$ are the signals at the *m*-th and *n*-th measurements for the probes of the non-phase shift and π-phase shift, respectively, $L_{F}$ is fiber length, v is light velocity in the fiber, $\omega_{B}\left(z\right)$ is the Brillouin frequency shift at distance z and $R_{m}\left(t,z\right)$ and $R_{n}\left(t,z\right)$ are spontaneous Brillouin backscatter coefficients of the *m*-th and *n*-th measurements. The spontaneous Brillouin scattering occurs due to density fluctuation by thermal agitation independently in the different segments of the fiber. It is well known that the auto-correlation of spontaneous Brillouin scattering has a Laplace distribution. Since the coefficient $R_{m}\left(t,z\right)$ can have the same property as the spontaneous Brillouin scattering, its auto-correlation may be given by

(6) $$AC\left(\tau ,u\right)=\underset{N\to \infty }{\lim}\frac{1}{N}\overset{N}{\underset{m=1}{\sum }}\left[\int_{-\infty }^{\infty }R_{m}\left(t,z\right)R_{m}{}^{*}\left(t+\tau ,z+u\right)dz\right]=Ae^{-\Gamma_{a}\left(z\right)\left|\tau \right|}\delta \left(u\right),$$

where N is the number of the repetitive measurements for time averaging, A is a proportional constant, $\Gamma_{a}$ is related to the phonon decay rate $\Gamma_{B}$ [19] as $\Gamma_{a}=\Gamma_{B}/2$, and δ(u) is the Dirac delta function and * denotes the complex conjugate.

Now, we will extract partial data of the Brillouin signals, $b_{m,0}\left(t\right)$ and $b_{n,\pi }\left(t\right)$, by using a pair of window functions $w_{L}\left(t-t_{0}\right)$ and $w_{S}\left(t-t_{0}\right)$ for each. As shown in Figure 2c, they have the same durations as the long and short probe pulse, *T_L_* and *T_S_*, and the same interval *T_I_*; $w_{L}\left(t-t_{0}\right)$ and $w_{S}\left(t-t_{0}\right)$ begin at $t=t_{0}$ and $t=t_{0}+T_{L}+T_{I}$, respectively. Then, sampled signals can be given by

(7) $$b_{q,m,p}\left(t,t_{0}\right)=b_{m,p}\left(t\right)w_{q}\left(t-t_{0}\right),$$

where the suffix p denotes 0 or π, and q denotes *L* or *S*.

Next, as explained in Section 1, we will consider cross-correlations between the backscatter signals sampled by $w_{L}\left(t-t_{0}\right)$ and $w_{S}\left(t-t_{0}\right)$. For the case of the backscatter due to the 0-shift probe pulse, we define the cross-correlation as follows:

(8) $$CC_{0}\left(\tau ,t_{0}\right)=\frac{1}{N}\sum_{m=1}^{N}\left[\frac{1}{T_{R}}\int_{-T_{R}/2}^{T_{R}/2}b_{L,m,0}\left(t,t_{0}\right)b_{S,m,0}{}^{*}\left(t+\tau ,t_{0}\right)dt\right].$$

where $T_{R}$ is the repetition period of the probe pulse. We assume N is so large that the statistical fluctuation of the thermally excited Brillouin backscatter can be ignored. Then, if we substitute Equation (7) into (8), we obtain

(9) $$CC_{0}\left(\tau ,t_{0}\right)=\frac{A}{T_{R}}\overset{L_{F}}{\underset{0}{\int }}\left\{e^{-\Gamma_{a}\left(z\right)\left|\tau \right|-j\omega_{B}\left(z\right)\tau }\left[\overset{T_{R}/2}{\underset{-T_{R}/2}{\int }}\left(p_{LL}+p_{LS}+p_{SL}+p_{SS}\right)dt\right]\right\}dz,$$

where

(10) $$\begin{array}{c} \left(\begin{array}{c} p_{LL}\\ \begin{array}{c} p_{LS}\\ p_{SL}\\ p_{SS} \end{array} \end{array}\right)=\left(\begin{array}{c} f_{L}\left(t-\frac{2z}{v}\right)f_{L}\left(t-\frac{2z}{v}+\tau \right)\\ f_{L}\left(t-\frac{2z}{v}\right)f_{S}\left(t-\frac{2z}{v}+\tau \right)\\ f_{S}\left(t-\frac{2z}{v}\right)f_{L}\left(t-\frac{2z}{v}+\tau \right)\\ f_{S}\left(t-\frac{2z}{v}\right)f_{S}\left(t-\frac{2z}{v}+\tau \right) \end{array}\right)\\ \times w_{L}\left(t-t_{0}\right)w_{S}\left(t-t_{0}+\tau \right). \end{array}$$

Similar consideration for the case of the π–shift probe pulse yields the other cross-correlation. Since Equation (3) for the case of the π–shift probe pulse differs only in the sign of $f_{S}\left(t\right)$ and the initial phase of the optical field from Equation (2), and since the initial phase does not affect the cross-correlation, we can obtain the cross-correlation for the case of the π-shift probe pulse just by replacing $f_{S}\left(\cdot \right)$ of the cross-correlation for the non-phase-shift case with $-f_{S}\left(\cdot \right)$. The result is given by

(11) $$CC_{\pi }\left(\tau ,t_{0}\right)=\frac{A}{T_{R}}\overset{L_{F}}{\underset{0}{\int }}\left\{e^{-\Gamma_{a}\left(z\right)\left|\tau \right|-j\omega_{B}\left(z\right)\tau }\left[\overset{T_{R}/2}{\underset{-T_{R}/2}{\int }}\left(p_{LL}-p_{LS}-p_{SL}+p_{SS}\right)dt\right]\right\}dz.$$

with Equation (10). By inspecting the product term $p_{SL}$, we notice that $p_{SL}=0$. This is because $p_{SL}$ expresses the product of the backscatter of the short pulse sampled with the long window function and the backscatter of the long pulse sampled with the short window function. However, these samplings never occur simultaneously, since we set both the long pulse and the long window function in front of the short ones, respectively, as shown in Figure 2.

Next, we define the differential cross-correlation as

(12) $$DCC\left(\tau ,t_{0}\right)=CC_{0}\left(\tau ,t_{0}\right)-CC_{\pi }\left(\tau ,t_{0}\right).$$

Substituting Equations (9–11) into (12) and considering $p_{SL}=0$ yields

(13) $$DCC\left(\tau ,t_{0}\right)=\frac{2A}{T_{R}}\int_{0}^{L_{F}}\left\{e^{-\Gamma_{a}\left(z\right)\left|\tau \right|-j\omega_{B}\left(z\right)\tau }\left[\int_{-T_{R}/2}^{T_{R}/2}p_{LS}dt\right]\right\}dz.$$

In the following, for simplicity, we will assume $f_{L}\left(t\right)$ and $f_{S}\left(t\right)$ have the same amplitude ($E_{L}=E_{S}=E_{in}$) and will use rectangular functions for expressing both the pulses and the window functions as follows:

(14) $$f_{L}\left(t\right)/E_{in}=w_{L}\left(t\right)=\mathrm{rect}\left(t,T_{L}\right),$$

(15) $$f_{S}\left(t\right)/E_{in}=w_{S}\left(t\right)=\mathrm{rect}\left(t-T_{L}-T_{I},T_{S}\right),$$

where

(16) $$\mathrm{rect}\left(t,T\right)=\{\begin{array}{c} \begin{array}{cc} 1 & 0<t<T \end{array}\\ \begin{array}{cc} 0 & t\leq 0,t\geq T \end{array} \end{array}.$$

Then, Equation (13) is given by:

(17) $$DCC\left(\tau ,t_{0}\right)=\frac{2AE_{in}{}^{2}}{T_{R}}\int_{0}^{L_{F}}\left\{e^{-\Gamma_{a}\left(z\right)\left|\tau \right|-j\omega_{B}\left(z\right)\tau }W\left(\tau ,z\right)\right\}dz.$$

where

(18) $$\begin{array}{c} W\left(\tau ,z\right)=\int_{-T_{R}/2}^{T_{R}/2}[\mathrm{rect}\left(t-\frac{2z}{v},T_{L}\right)\mathrm{rect}\left(t-\frac{2z}{v}-T_{L}-T_{I}+\tau ,T_{S}\right).\\ \times \mathrm{rect}\left(t-t_{0},T_{L}\right)\mathrm{rect}\left(t-t_{0}-T_{L}-T_{I}+\tau ,T_{S}\right)]dt. \end{array}$$

By inspecting the product $\mathrm{rect}\left(t-\frac{2z}{v}-T_{L}-T_{I}+\tau ,T_{S}\right)\mathrm{rect}\left(t-t_{0}-T_{L}-T_{I}+\tau ,T_{S}\right)$ in Equation (18), W(τ,z) is set in a narrow region of $\left|\delta z\right|=\left|z-z_{0}\right|<\Delta z$, where $z_{0}=vt_{0}/2$ and $\Delta z=vT_{S}/2$. Outside the region, W(τ,z)=0. Therefore, we may replace $\omega_{B}\left(z\right)$ and $\Gamma_{a}\left(z\right)$ in Equation (17) with constant values of $\omega_{B}\left(z_{0}\right)$ and $\Gamma_{a}\left(z_{0}\right)$.

For $\left|\delta z\right|=\left|z-z_{0}\right|<\Delta z$, W(τ,z) is given as follows:

(19) $$W\left(\tau ,z\right)=\{\begin{array}{cc} 0 & \tau \leq T_{I}+\left(2/v\right)\left|\delta z\right|\\ \tau -T_{I}-\left(2/v\right)\left|\delta z\right| & T_{I}+\left(2/v\right)\left|\delta z\right|\leq \tau \leq T_{I}+T_{S}\\ T_{S}-\left(2/v\right)\left|\delta z\right| & T_{I}+T_{S}\leq \tau \leq T_{I}+T_{L}\\ -\left(\tau -T_{I}\right)-\left(2/v\right)\left|\delta z\right|+T_{L}+T_{S} & T_{I}+T_{L}\leq \tau \leq T_{I}+T_{L}+T_{S}-\left(2/v\right)\left|\delta z\right|\\ 0 & \tau \geq T_{I}+T_{L}+T_{S}-\left(2/v\right)\left|\delta z\right| \end{array}$$

**Figure 3:**
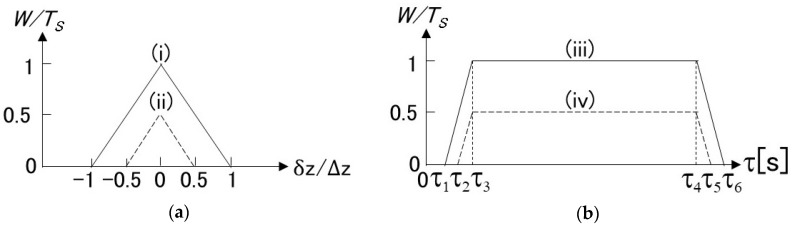
Function W(τ,z): (**a**) z-dependency, (i) $\tau_{3}\leq \tau \leq \tau_{4}$ [s], (ii) $\tau =\tau_{2},\tau =\tau_{5}$ [s]; (**b**) τ –dependency, (iii) δz=0 [m], (iv) |δz|=Δz/2 [m]; $\tau_{1}=T_{I}$ [s], $\tau_{2}=T_{I}+\left(T_{S}/2\right)$ [s], $\tau_{3}=T_{I}+T_{S}$ [s], $\tau_{4}=T_{I}+T_{L}$ [s], $\tau_{5}=T_{I}+T_{L}+\left(T_{S}/2\right)$ [s], $\tau_{6}=T_{I}+T_{L}+T_{S}$ [s].

Figure 3a shows the relationship between W(τ,z) and the distance z. We can see from Figure 3a that $W\left(\tau ,z\right)\approx T_{S}$ in the narrow region of |δz|≤Δz/2.

Figure 3b shows the relationship between W(τ,z) and the lag τ. We can see from Figure 3b that $W\left(\tau ,z\right)\approx T_{S}$ in the wide region of τ from $\tau_{2}=T_{I}+\left(T_{S}/2\right)$ to $\tau_{5}=T_{I}+T_{L}+\left(T_{S}/2\right)$.

Based on Figure 3a,b and Equation (19), we can further approximate Equation (17) as

(20) $$DCC\left(\tau ,t_{0}\right)\approx \frac{2AE_{in}{}^{2}}{T_{R}}\Delta zT_{s}e^{-\Gamma_{a}\left(z_{0}\right)\left|\tau \right|-j\omega_{B}\left(z_{0}\right)\tau }\mathrm{rect}\left(\tau -\tau_{2},\tau_{5}-\tau_{2}\right).$$

As defined in Equation (16), $\mathrm{rect}\left(\tau -\tau_{2},\tau_{5}-\tau_{2}\right)$ is a rectangular function in the interval ($\tau_{2}$, $\tau_{5}$) with the difference $\tau_{5}-\tau_{2}=T_{L}$.

From both Figure 3a,b and Equations (17–19), we can confirm that the function W(τ,z) plays a role of the window function multiplied to $e^{-\Gamma_{a}\left(z_{0}\right)\left|\tau \right|-j\omega_{B}\left(z_{0}\right)\tau }$ and that it has long and short intervals in the τ-axis and in the z-axis, respectively, as we expected.

### 2.3. Spatial Resolution

As described above, $p_{LS}$ and W(τ,z) center on $z=z_{0}$ and effectively range from $z=z_{0}-vT_{S}/4$ to $z=z_{0}+vT_{S}/4$, as shown in Figure 3a. Thus, we can obtain the localized cross-correlation from Equation (17) and define the spatial resolution Δz of PSP-BOTDR as

(21) $$\Delta z=vT_{S}/2.$$

This formula of the spatial resolution is the same as that for the classic BOTDR using a single probe pulse of duration $T_{S}$.

### 2.4. Spectral Analysis

We can obtain the complex spectrum of the localized Brillouin backscatter by applying the Fourier transform to $DCC\left(\tau ,t_{0}\right)$ with respect to lag τ:

(22) $$B\left(\omega \right)={ℱ}_{\tau }\left[DCC\left(\tau ,t_{0}\right)\right].$$

If we use $DCC\left(\tau ,t_{0}\right)$ from Equation (20), B(ω) is approximately given by

(23) $$B\left(\omega \right)\approx \frac{2AE_{in}{}^{2}}{T_{R}}\Delta zT_{S}\frac{1}{-j\left(\omega -\omega_{B}\right)+\Gamma_{a}}e^{\left[-\Gamma_{a}+j\left(\omega -\omega_{B}\right)\right]\left(T_{I}+T_{S}/2\right)}\left\{1-e^{\left[-\Gamma_{a}+j\left(\omega -\omega_{B}\right)\right]T_{L}}\right\},$$

where $\omega_{B}=\omega_{B}\left(z_{0}\right)$ and $\Gamma_{a}=\Gamma_{a}\left(z_{0}\right)$. It should be noted that in the derivation of Equation (23) the range of the Fourier integral does not include the negative τ region since W(τ,z)=0 for τ≤0.

The absolute value of B(ω) is given by

(24) $$B_{a}\left(\omega \right)=\left|B\left(\omega \right)\right|\approx B_{0}e^{-\Gamma_{a}\left(T_{I}+T_{S}/2\right)}\sqrt{\frac{1+e^{-2\Gamma_{a}T_{L}}-2e^{-\Gamma_{a}T_{L}}\cos\left(\omega -\omega_{B}\right)T_{L}}{{\left(\omega -\omega_{B}\right)}^{2}+\Gamma_{a}{}^{2}}},$$

where $B_{0}=2AE_{in}{}^{2}\Delta zT_{S}/T_{R}$. Since the spectrum |B(ω)| is maximum when $\omega =\omega_{B}$, we can find the BFS by analyzing the peak of the spectrum. However, the spectrum width is rather broad compared to that of the intrinsic BSS. If we assume for simplicity that $\Gamma_{a}\left(T_{I}+T_{S}/2\right)\ll 1$ and $\Gamma_{a}T_{L}\ll 1$, the width (FWHM) of the |B(ω)| is given by

(25) $$\Delta \omega =2\sqrt{3}\Gamma_{a},$$

which should be much narrower than $1/T_{S}$, but $\sqrt{3}$ times broader than that of the intrinsic BSS, $\Gamma_{B}=2\Gamma_{a}$ [18].

Another spectral analysis method is to use a real part of B(ω): Br(ω)=Re[B(ω)]. If we assume again for simplicity that $\Gamma_{a}\left(T_{I}+T_{S}/2\right)\ll 1$ and $\Gamma_{a}T_{L}\ll 1$, the spectrum $B_{r}\left(\omega \right)$ is given by

(26) Br(ω)=Re[B(ω)]≈B0Γa(ω−ωB)2+Γa2,

which has the same profile as the intrinsic BSS. Therefore, we should obtain an improved frequency resolution by using $B_{r}\left(\omega \right)$ rather than $B_{a}\left(\omega \right)$.

It is also possible to use an imaginary part of B(ω) for the spectral analysis Bi(ω)=Im[B(ω)]. Provided that we use the same conditions above, $B_{i}\left(\omega \right)$ is given by

(27) Bi(ω)=Im[B(ω)]≈B0(ω−ωB)(ω−ωB)2+Γa2.

Based on Equation (27), the BFS can be obtained by seeking the zero-crossing of $B_{i}\left(\omega \right)$. Since the $B_{i}\left(\omega \right)$ rapidly increases from the negative minimum to the positive maximum within the narrow range of $2\Gamma_{a}$ width, a good frequency resolution similar to that based on $B_{r}\left(\omega \right)$ can be expected.

The validity of the analysis based on $B_{r}\left(\omega \right)$ and $B_{i}\left(\omega \right)$ will be verified by the experiments presented in Section 3.

### 2.5. Signal Processing

We will reconsider the Fourier transform of the differential cross-correlation, expressed by Equation (22), as follows:

(28) $$B\left(\omega \right)={ℱ}_{\tau }\left[DCC\left(\tau ,t_{0}\right)\right]={ℱ}_{\tau }\left[CC_{0}\left(\tau ,t_{0}\right)\right]-{ℱ}_{\tau }\left[CC_{\pi }\left(\tau ,t_{0}\right)\right].$$

If we introduce Equation (8) for the 0-shift probe pulse and its counterpart for the π–shift probe pulse into Equation (28) and use a cross-correlation theorem, we obtain

(29) $$B\left(\omega \right)=\frac{1}{N}\overset{N}{\underset{m=1}{\sum }}\left\{{ℱ}_{t}\left[b_{L,m,0}\left(t,t_{0}\right)\right]{ℱ}_{t}{}^{*}\left[b_{S,m,0}\left(t,t_{0}\right)\right]-{ℱ}_{t}\left[b_{L,m,\pi }\left(t,t_{0}\right)\right]{ℱ}_{t}{}^{*}\left[b_{S,m,\pi }\left(t,t_{0}\right)\right]\right\},$$

where ${ℱ}_{t}$ denotes the Fourier transform with respect to t. Therefore, we can also estimate B(ω) from the average of the differential cross-spectrum. Equation (29) is useful to efficiently process the backscatter data and obtain the BSS by utilizing high-performance FFT processors.

## 3. Results

### 3.1. Experimental Setup

The experimental setup in our work was the same as in [12]. A pair of probe pulses consisted of a long pulse and a short pulse each, as expressed by Equations (2) and (3). The duration $T_{L}$ of the long pulse was set to 10, 20, 30 and 60 ns to investigate the frequency resolution dependence on the duration $T_{L}$. The duration $T_{S}$ of the short pulse was set to 2 ns to attain 20-cm spatial resolution. The interval time $T_{I}$ could theoretically be zero; however, it takes about 0.3 ns for our Mach–Zehnder modulator to transiently provide the π–shift for the short probe pulse, while no transient time exists for the 0-shift probe pulse. This imbalance could destroy the subtraction principle based on Equations (12) and (13) of PSP-BOTDR. Thus, we set $T_{I}$ to 0.5 ns for the pair of probes [12]. The attenuation of the differentiate cross-correlation due to the interval can be ignored since the factor of the attenuation is estimated at $e^{-\Gamma_{a}T_{I}}=0.95$ by assuming a typical BSS linewidth of $\Gamma_{B}/2\pi =\Gamma_{a}/\pi =30$ MHz and $T_{I}=0.5$ ns. The peak power of the probe was set to 300 mW for both long and short pulses. The fiber under test was a 330-m single-mode fiber including 30 cm and 3 m sections that differed in the BFS by about 50 MHz from the other sections. The short sections were about 310 and 320 m from the input end of the fiber under test. The number of repetitions for averaging N was 50,000 for each probe pulse case. All data were saved on a digital oscilloscope and sent to a personal computer (PC). We obtained the spectrum by computing Equation (29). The Fourier transforms were executed by applying the FFT to the data. Lorentz curve fitting was used to obtain BFS from the spectra of $B_{a}\left(\omega \right)$ and $B_{r}\left(\omega \right)$, while linear fitting was used for the spectrum of $B_{i}\left(\omega \right)$.

### 3.2. Experimental Results and Discussions

**Figure 4:**
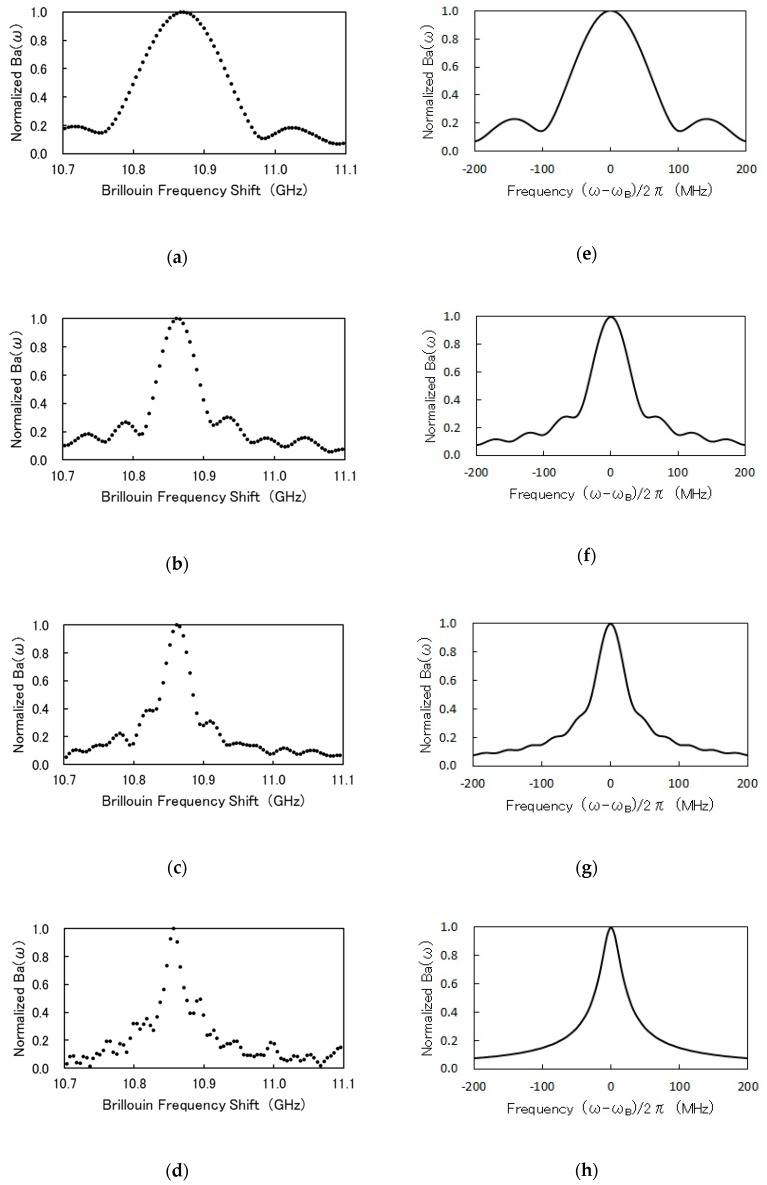
$B_{a}\left(\omega \right)$ for various durations of $T_{L}$: $T_{S}=2$ ns and $T_{I}=0.5$ ns; (**a**–**d**): experiments; (**e**–**h**): calculations; $T_{L}=$ 10 ns for (**a**,**e**); $T_{L}=$ 20 ns for (**b**,**f**); $T_{L}=$ 30 ns for (**c**,**g**); $T_{L}=$ 60 ns for (**d**,**h**).

Figure 4a–d shows the experimental spectra of normalized $B_{a}\left(\omega \right)$ for durations of $T_{L}$=10, 20, 30 and 60 ns, while Figure 4e–h illustrates numerical spectra corresponding to Figure 4a–d. The calculations assumed that $\Gamma_{a}/\pi =30$ MHz. We can see that, on the whole, the experimental and numerical spectra agreed well with each other. Both experiments and calculations showed that the spectrum width decreases as the duration $T_{L}$ increases. Therefore, we could expect that the BFS resolution would be improved with an increase in $T_{L}$. Accordingly, the BFS resolutions were evaluated by calculating the standard deviations of the BFSs in the region from 200 to 300 m of the fiber under test. The experimental results of the width and the BFS resolution are summarized in Table 1.

The BFS resolution for $T_{L}=$10 ns was 3.4 MHz, which was poorer than the resolutions of 2.2 MHz for $T_{L}=$ 20 ns and 2.7 MHz for $T_{L}=$30 ns, as expected. However, the BFS resolution for $T_{L}$ = 60 ns deteriorated to 4.0 MHz. This was probably caused by the degradation of signal-to-noise ratio due to the difference in the rate of increase of signal and noise with the increase of $T_{L}$. The integration of the differential cross-correlation $DCC\left(\tau ,t_{0}\right)$ of the backscatter with respect to the lag τ may increase with $T_{L}$ but reach a constant value for long $T_{L}$. This is due to the fact that $DCC\left(\tau ,t_{0}\right)$ for the backscatter is almost zero for large τ. In contrast, the integration of the variations in the cross-correlation of noise itself and in the cross-correlation between noise and backscatter may continue to increase with $T_{L}$. Measured $B_{a}\left(\omega \right)$ for $T_{L}$ = 60 ns was noisy, as shown in Figure 4d. Thus, we found that a choice of $T_{L}$ = 20–30 ns yields better BFS resolution. This is probably because this choice improves the trade-off relation of the differential cross-correlation of the backscatter signal and the reduction in the cross-correlations related to the above noise.

**Figure 5:**
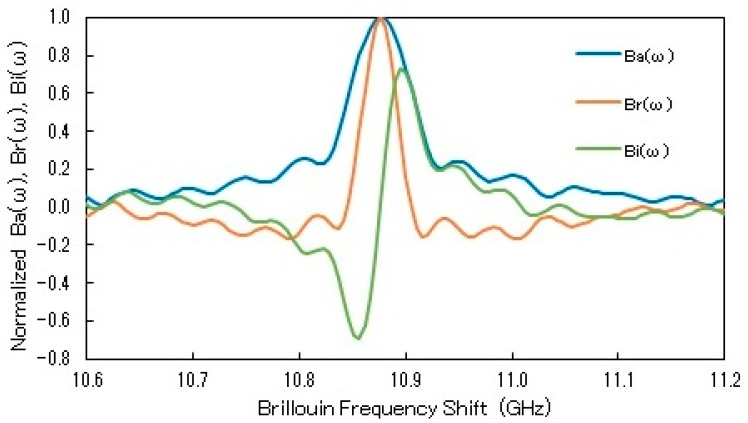
Comparisons of experimentally obtained spectra, $B_{a}\left(\omega \right)$, $B_{r}\left(\omega \right)$ and $B_{i}\left(\omega \right)$, drawn by blue, orange and green curves, respectively; $T_{L}=20$ ns, $T_{S}=2$ ns, $T_{I}=0.5$ ns.

Figure 5 shows experimentally obtained spectra, $B_{a}\left(\omega \right)$, $B_{r}\left(\omega \right)$ and $B_{i}\left(\omega \right)$, obtained using the probe pulse pair of $T_{L}$ = 20 ns, $T_{S}=2$ ns and $T_{I}=0.5$ ns. The widths (FWHM) of $B_{a}\left(\omega \right)$ and $B_{r}\left(\omega \right)$ were 69 and 34 MHz, respectively; the former was 2.0 times broader than the latter, in relatively good agreement with the theoretical value of $\sqrt{3}$. The frequency difference between the maximum and minimum of $B_{i}\left(\omega \right)$ was 43 MHz. This frequency separation was a little larger than the width of $B_{r}\left(\omega \right)$, but was much less than the width of $B_{a}\left(\omega \right)$. Based on the results, we can expect better BFS resolution by using $B_{r}\left(\omega \right)$ and $B_{i}\left(\omega \right)$ rather than $B_{a}\left(\omega \right)$.

**Figure 6:**
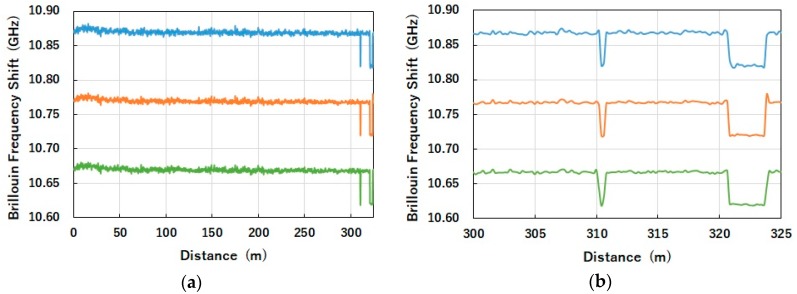
Brillouin frequency shift (BFS) distributions evaluated using three spectra, $B_{a}\left(\omega \right)$, $B_{r}\left(\omega \right)$ and $B_{i}\left(\omega \right)$, shown by blue, orange and green curves, respectively. Frequency offset is added to orange and green curves for ease of viewing. (**a**) Whole map, (**b**) expanded view; $T_{L}=20$ ns, $T_{S}=2$ ns, $T_{I}=0.5$ ns.

Finally, Figure 6 shows the BFS distributions measured over the single-mode fiber under testing. The BFS distributions were evaluated using three spectra each, $B_{a}\left(\omega \right)$, $B_{r}\left(\omega \right)$ and $B_{i}\left(\omega \right)$, which were plotted by blue, orange and green curves, respectively. In each curve, we can see a change of 50 MHz in the BFS over the 30 cm and 3 m sections near the end of the fiber with a spatial resolution of 20 cm. To accurately evaluate the BFS resolution, the standard deviations of BFSs in the region from 311 to 320 m of the fiber were calculated. This was due to the fact that the fiber in the region was carefully coiled with a diameter of up to 30 cm, so that no accidental strain would occur. Evaluated BFS resolutions were 1.5, 1.1 and 1.2 MHz for the spectra of $B_{a}\left(\omega \right)$, $B_{r}\left(\omega \right)$ and $B_{i}\left(\omega \right)$, respectively. Thus, we confirmed that the spectra of $B_{r}\left(\omega \right)$ and $B_{i}\left(\omega \right)$ provide better BFS resolution than $B_{a}\left(\omega \right)$, which was used previously.

## 4. Discussion

We have derived an expression for the complex spectrum obtained by the high-spatial-resolution BOTDR called PSP-BOTDR, and have explained the principle of PSP-BOTDR theoretically. Based on the expression, we have also proposed the use of new spectra—a real part and an imaginary part of the complex spectrum. We have theoretically and experimentally clarified that the newly proposed spectra of the real and imaginary parts of the spectrum have sharper profiles than the absolute of the complex spectrum that was used previously. Our experiments have also clarified that the new spectra provide better BFS resolution than the previous one. As in a BOTDR approach that combines the FFT technique and the monopolar complementary code technique [20], bipolar code can also be applied to the PSP-BOTDR to improve the BFS resolution and make faster measurements; we have reported its initial success in operating the 20-cm spatial resolution coded PSP-BOTDR [21]. We believe the results reported in this article will help to significantly improve the performance of such high- spatial-resolution BOTDRs.

Finally, we would like to note that this article discusses the basic property of PSP-BOTDR based on spontaneous Brillouin scattering. However, an increase in the peak power of the probe pulse and the coding of the probe pulse with a long code to extend the measurement range would manifest the SBS; therefore, we should take into account the SBS. The mathematical formalism of the SBS that affects PSP-BOTDR and experiments involving this will constitute our future work.

## Figures and Tables

**Table 1:** Experimental results of Brillouin spectrum width and Brillouin frequency shift (BFS) resolution.

$T_{L}$ (ns)	Width (FWHM) (MHz)	BFS Resolution (MHz)
10	137	3.4
20	66	2.2
30	46	2.4
60	36	4.0
